# Predictive Power of the Albumin–Bilirubin Score for Hepatotoxicity in Stereotactic Ablative Radiation Therapy for Hepatocellular Carcinoma

**DOI:** 10.3390/cancers15153777

**Published:** 2023-07-25

**Authors:** Jihyeon Joo, Hosang Jeon, Dongwoon Kim, Wontaek Kim, Jiho Nam, Donghyun Kim, Dahl Park, Choongrak Kim, Yongkan Ki

**Affiliations:** 1 Department of Radiation Oncology, Pusan National University School of Medicine, Yangsan 50612, Republic of Korea; hi_juji@daum.net (J.J.);; 2 Department of Radiation Oncology, Pusan National University Yangsan Hospital, Yangsan 50612, Republic of Korea; 3 Department of Radiation Oncology, Pusan National University Hospital, Busan 49241, Republic of Korea; 4 Department of Statistics, Pusan National University, Busan 46241, Republic of Korea

**Keywords:** albumin, bilirubin, prognosis, ALBI grade

## Abstract

**Simple Summary:**

The albumin–bilirubin (ALBI) score has been proposed as an alternative to the Child-Turcotte-Pugh (CTP) classification for assessing liver function. We evaluated the prognostic performance of the ALBI score and determined the optimal cutoff point as a potential predictor of hepatotoxicity after stereotactic body radiation therapy. To compare ALBI and CTP scores, receiver operating characteristic (ROC) curves were calculated, and DeLong’s test was used for statistical analysis. The optimal ALBI score cutoff was calculated. The baseline ALBI score was an independent prognostic factor for hepatotoxicity. The optimal ALBI cutoff value to predict CTP increase was −2.47, which is higher than the widely accepted reference point for ALBI grades 1 and 2. The ALBI score had greater predictive power compared to the CTP score.

**Abstract:**

Assessment of liver function is crucial in predicting treatment outcomes for hepatocellular carcinoma (HCC). This study aimed to evaluate the prognostic performance of the albumin–bilirubin (ALBI) score for predicting hepatotoxicity following stereotactic body radiation therapy (SBRT) in HCC patients. A retrospective analysis was conducted on 123 HCC cases treated between 2018 and 2020. ALBI and Child-Turcotte-Pugh (CTP) scores were calculated, and hepatotoxicity was defined as a post-SBRT CTP score increase ≥2. Receiver operating characteristic (ROC) curves were used for comparison. The optimal cutoff value of the ALBI score was determined. Among the 121 patients analyzed, hepatotoxicity occurred in 5%. The ALBI score showed better predictive accuracy (area under the ROC curve: 0.77) than the CTP score. The optimal cutoff value of the ALBI score was −2.47, with a sensitivity of 85.7% and a specificity of 71.1%. Multivariable analysis revealed that ALBI score and PTV were significant factors for hepatotoxicity. In conclusion, the ALBI score demonstrated prognostic value for hepatotoxicity prediction after SBRT in HCC patients. Considering the ALBI score and PTV provides valuable insights for assessing hepatotoxicity risk during SBRT treatment for HCC.

## 1. Introduction

Stereotactic body radiation therapy (SBRT) has been demonstrated to be an effective treatment option with high rates of local control in patients with hepatocellular carcinoma (HCC), particularly for those who are not suitable candidates for resection or radiofrequency ablation [1,2,3]. Despite the efficacy of SBRT in treating HCC, most patients with HCC have underlying liver cirrhosis or other liver dysfunctions, which significantly impact the treatment outcomes [4]. Therefore, evaluating the patient’s liver function is a critical component of both staging systems and treatment guidelines, such as the Barcelona Clinic Liver Cancer (BCLC) staging system. Similarly, in the context of SBRT for HCC, the accurate prediction of potential changes in liver function is crucial for making informed treatment decisions and optimizing SBRT planning.

The Child-Turcotte-Pugh (CTP) grading system is currently the most widely accepted method to assess liver function in patients with liver disease. The CTP classification system incorporates several parameters, such as serum albumin and bilirubin levels, coagulation factors, and the presence and severity of ascites and hepatic encephalopathy [5,6]. However, clinical assessment of ascites and hepatic encephalopathy may be subjective and difficult to consistently score by different evaluators. Recently, the ALBI grade has emerged as a novel scoring system suggested as a substitute for the CTP classification in evaluating liver function [7]. With the introduction of the ALBI score, studies have shown the advantage of the ALBI score as an assessment and treatment plan for HCC and chronic liver disease compared to the CTP score. The superiority of ALBI grade in predicting overall survival and hepatic function has been demonstrated in hepatic resection, radiofrequency ablation (RFA), SBRT, and transarterial chemoembolization (TACE) [8,9,10,11,12,13]. However, the ALBI grade cutoff value validation in patients with HCC undergoing SBRT remains inadequate, as only a limited number of retrospective studies have addressed the optimal cutoff value for this treatment modality [10].

This study aimed to evaluate the prognostic performance of the ALBI score and determine the optimal cutoff value as a potential predictor of hepatotoxicity after SBRT.

## 2. Materials and Methods

### 2.1. Patients

This study has received a waiver of Institutional Review Board review. The eligibility criteria were as follows: (1) diagnosis of HCC based on histologic or radiologic criteria; (2) CTP class A or B liver disease; and (3) adequate residual functional liver volume. In total, 123 patients with primary or recurrent HCCs treated with SBRT at a single institution were retrospectively reviewed between 1 September 2018 and 31 December 2020.

### 2.2. ALBI Score

The equation for the ALBI score calculation was (log10 bilirubin × 0.66) + (albumin × −0.085), where bilirubin and albumin were in μmol/L and g/L, respectively [7]. The following cutoff points were used to define the three prognostic groups: ALBI grade 1 (≤−2.60), ALBI grade 2 (>−2.60 to ≤−1.39), and ALBI grade 3 (>−1.39).

### 2.3. Treatment

For RT planning, a 4-dimensional computed tomography (CT) simulation was performed (GE LightSpeed RT 16; GE Healthcare, Chicago, IL, USA) with abdominal compression to minimize respiratory motion. The CT slice thickness was set at 5 mm. CT series were sorted according to respiratory phase using 4-dimensional imaging software (AW 4.7; GE Healthcare, North Richland, TX, USA). The gross tumor volume (GTV) was defined as the visible HCC in each respiratory phase and then summed to create the internal target volume. The planning target volume (PTV) margin was 5 mm in each direction, around the internal target volume. Contouring and treatment planning were performed using a radiation therapy planning system (Monaco™; Elekta, Stockholm, Sweden). The recommended prescription dose was 48 Gy and was individualized to achieve a normal liver volume receiving less than 15 Gy (rV15Gy) > 700 mL. The treatment was delivered in four fractions on alternate days with daily image guidance using a linear accelerator (Versa HD; Elekta, Stockholm, Sweden).

### 2.4. Evaluation

After the completion of SBRT, all patients were evaluated within 3 months. Contrast-enhanced CT and/or MRI, complete blood counts, coagulation tests, liver function tests, alpha-fetoprotein, and PIVKA-II levels were routinely examined. Hepatotoxicity was defined as an increase in the CTP score ≥ 2 within 3 months of SBRT completion compared to that before treatment and without evidence of HCC progression, according to the Response Evaluation Criteria in Solid Tumors, version 1.1.

### 2.5. Statistics

Patient characteristics were summarized using means, medians, standard deviations, and proportions as appropriate. Proportions were tested using the χ^2^ test or Fisher’s exact test. Pearson’s correlation test was used to measure the existence of a correlation between two variables, and the Student’s *t*-test was used to compare means between two groups. DeLong’s test was used to compare the two receiver operating characteristic (ROC) curves. A logistic regression model was used for the univariate or multivariate analyses. Overall survival (OS) was defined from the date of SBRT initiation until the date of death at the last follow-up. A Kaplan–Meier curve with a log-rank test was used to estimate the OS rates for different groups. Statistical significance was defined as a *p*-value < 0.05.

## 3. Results

### 3.1. Patients and Treatment Characteristics

In two patients, toxicity development was diagnosed on the same day as tumor progression. In both, the extent of progression was massive, a confounding factor for toxicity. Following the exclusion of these two patients, the data from 121 patients were analyzed. The median follow-up time was 15.5 months (interquartile range, 9.7–22.1 months). The clinical characteristics of the patients are summarized in Table 1. This study included 100 male and 21 female patients, with a mean age at treatment of 67.9 years. There were 93 patients with a CTP score of 5, 19 with a CTP score of 6, 4 with a CTP score of 7, and 5 with a CTP score of 8. The median ALBI score was −2.6 ± 0.4. There were 66 patients with ALBI grade 1, 54 with ALBI grade 2, and one with ALBI grade 3. The ALBI grade and CTP class correlated well (*p* < 0.001, Fisher’s exact test). Among patients with CTP-A disease, 66 had ALBI grade 1, and 46 had ALBI grade 2 disease. None of the patients had ALBI grade 3. None of the patients with CTP class B disease had ALBI grade 1, 8 had ALBI grade 2, and 1 had ALBI grade 3 disease. For statistical analysis, only one ALBI grade 3 patient was classified as ALBI grade 2. ALBI scores were significantly different for each CTP class (*p* < 0.001, *t*-test). The average ALBI score in CTP class A was −2.7 ± 0.3 (−2.8 ± 0.3 in CTP-A5; −2.1 ± 0.3 in CTP-A6) and −1.7 ± 0.3 in CTP class B disease (−1.7 ± 0.4 in CTP-B7; −1.8 ± 0.2 in CTP-B8). The ALBI and CTP scores were highly linearly correlated, with a Pearson’s correlation coefficient of 0.76 (*p* < 0.001). The mean liver dose (MLD) was 8.0 ± 2.9 Gy. The volume of the liver receiving 15 Gy (V15) was 1023.5 ± 249.6 mL. Constraint rV15 ≥ 700 mL was achieved in 117 (97%) patients and violated in 4 (3%) patients, ranging from 680–699 mL.

### 3.2. Predictors of Toxicity

**Figure 1:**
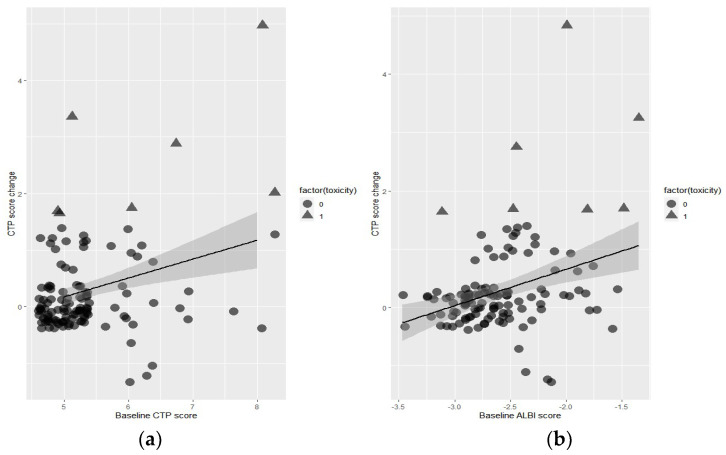
Correlation test between (**a**) baseline CTP score and CTP change after SBRT and (**b**) baseline ALBI score and CTP change after SBRT.

**Figure 2:**
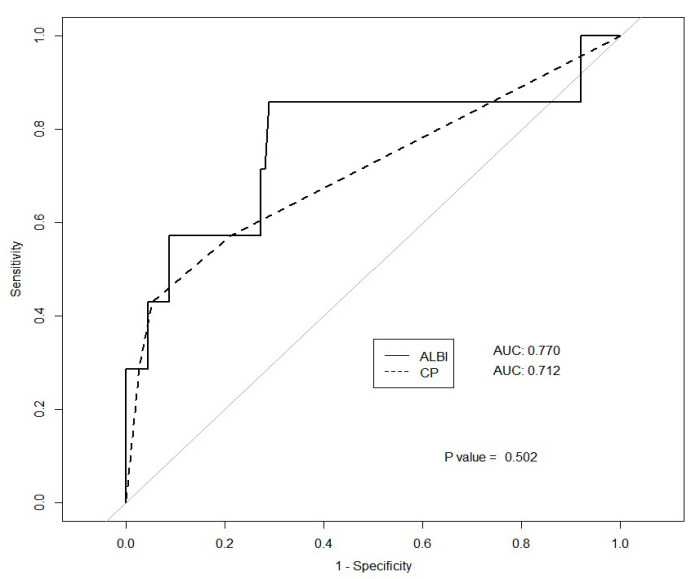
ROC curve for ALBI score and CTP score predicting hepatotoxicity.

Both baseline CTP and ALBI scores predicted post-SBRT CTP score changes. The correlation coefficients were 0.316 for the CTP score (*p* < 0.001) and 0.336 for the ALBI score (*p* < 0.001; Figure 1). A post-SBRT CTP score increase ≥ 2, defined as the occurrence of hepatotoxicity in this analysis, was observed in 7 patients (5%). The area under the ROC curve predicting hepatotoxicity for the ALBI score was 0.77, and that for the CTP score was 0.71 (*p* = 0.5019; Figure 2). The interval for the post-SBRT laboratory assessment had a median duration of 50 days (interquartile range, 28.5–60 days).

For hepatotoxicity occurrence, the following variables were tested: sex, age, platelet count, underlying liver disease (HBV vs. HCV vs. others), CTP score and class, ALBI score and grade, tumor size (cm), GTV (cc), PTV (cc), prescription dose, normal liver volume, mean liver dose (MLD), and rV15. In the univariate logistic regression analysis, the baseline CTP score and class, ALBI score and grade, and PTV were significant factors (Table 2). In the multivariable logistic regression, the ALBI score and PTV were significant predictors (odds ratio [OR] of 29.51 for ALBI score [95% CI 3.86–418.40], *p* = 0.003; OR 1.05 for PTV [95% CI 1.02–1.09], *p* = 0.004) (Table 2). All variables included in the multivariable model had VIFs < 10.0; thus, multicollinearity was not a concern.

### 3.3. Cutoff Value

**Figure 3:**
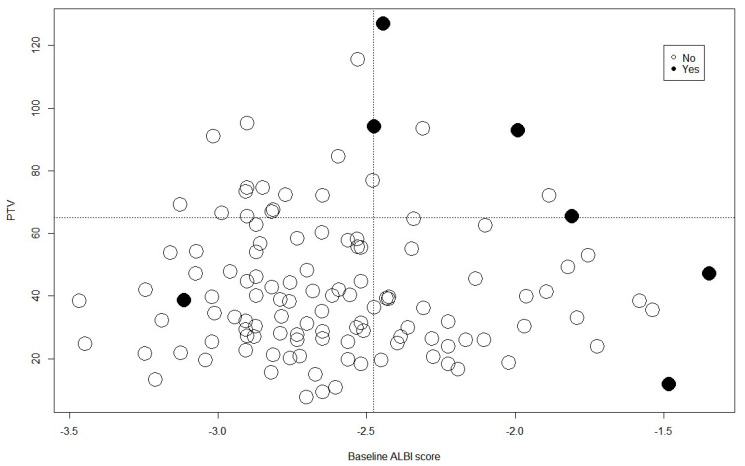
The incidence of hepatotoxicity in groups stratified by ALBI score of −2.47 and PTV of 65 mL.

The optimal cutoff value of the ALBI score for predicting hepatotoxicity was −2.47, with a sensitivity of 85.7% and a specificity of 71.1%. Of the patients with ALBI scores >−2.47, 15.38% (6 of 39) developed toxicity, compared with 1.22% (1 of 82) of patients with a score ≤−2.47. The cutoff value of the PTV was 65.32 mL, with a sensitivity of 57.1% and a specificity of 85.1%. Of the patients with PTV < 65 mL, 3.0% (3 of 100) developed toxicity, compared to 19.0% (4 of 21) in patients with PTV ≥ 65 mL. Patients were divided into the following four groups based on an ALBI score of 2.47 and a PTV of 65 mL: Group 1, ALBI score ≤−2.47 and PTV < 65 mL; Group 2, ALBI score ≤−2.47 and PTV ≥65; Group 3, ALBI score > −2.47 and PTV < 65; Group 4, ALBI score >−2.47 and PTV ≥65. The incidence of hepatotoxicity in Groups 1, 2, 3, and 4 was 1.47% (1 of 68), 6.25% (1 of 16), 6.25% (2 of 32), and 60% (3 of 5), respectively (*p* < 0.001; Figure 3).

### 3.4. Mean Liver Dose

**Figure 4:**
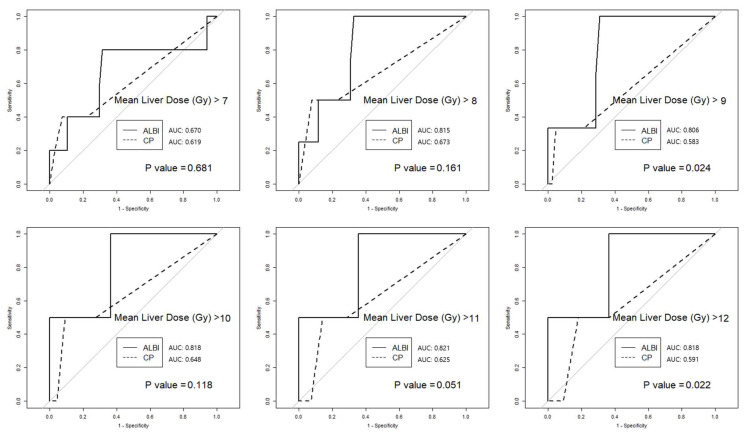
The predictive power of the ALBI score and CTP score was compared in different subgroups depending on the mean liver dose.

The predictive power of the ALBI and CTP scores was estimated in different subgroups, according to the MLD. Through MLD > 7 Gy, 8 Gy, 9 Gy, 10 Gy, 11 Gy, and 12 Gy, the AUC was higher for the ALBI score than for the CTP score. Significant differences were observed in groups with MLD >9 Gy, >11 Gy, and >12 Gy (Figure 4).

### 3.5. Survival

On univariate analysis for OS, the baseline CTP score, tumor size, and PIVKA-II were significant predictors. On multivariable analysis, CTP score and tumor size were significant predictors of inferior survival (HR of 2.793 for CTP score [95% CI 1.37–5.71], *p* = 0.005; OR of 2.184 for tumor size [95% CI 1.23–3.87], *p* = 0.007) (Table 3). The worsening of CTP and ALBI scores after SBRT was significantly correlated with a worse overall survival time. When these two factors were added to the multivariable analysis, CTP score change and tumor size were significant predictors of inferior survival (HR of 2.19 for CTP score change [95% CI 1.11–4.35], *p* = 0.025; HR of 2.22 for tumor size [95% CI 1.24–3.96], *p* = 0.007). In this study, 119 cases received CT, and 95 cases received MRI after 3 months of treatment. Among the post-SBRT MRI patients, 44 showed hypointense areas in the hepatobiliary phase. However, we found no significant relationship between these hypointense areas and the post-treatment changes in ALBI (*p* = 0.371 for ALBI grade and 0.090 for ALBI score).

## 4. Discussion

In the present study, the ALBI score was a more effective method for predicting post-SBRT early liver function decline compared to CTP. The optimal ALBI cutoff value to predict hepatotoxicity was −2.47, resulting in substantially different risks of CTP increase (1.22% vs. 15.38%). Pre-treatment CTP score, post-treatment CTP increase, and tumor size were significant prognostic factors for OS.

Our findings are consistent with previous analyses assessing the baseline ALBI score as a predictor of toxicity after liver SBRT [10,14,15,16,17]. Murray et al. investigated two prospective series of patients with HCC who received 6-fraction SBRT at the Princess Margaret Cancer Center [16]. The analysis of 102 patients revealed that the ALBI system was better than the CTP score for determining OS and toxicity. When the ALBI score was considered, the CTP score was not significant in multivariable models [16]. Toesca et al. analyzed 60 patients, 40 with HCC and 20 with cholangiocarcinoma, treated with SBRT, comparing the ALBI and CTP scores in the assessment of liver function [17]. The authors developed models to predict liver function decline based on the CTP or ALBI score and radiation dose. They found that the ALBI score-based model better explained liver function decline than the CTP model [17]. 

Of the 121 patients enrolled in our study, 112 were CTP class A, with a predominant CTP score of 5 in 83% of cases (93 patients). Within the CTP A group, 66 were classified as ALBI grade 1 and 56 as grade 2, revealing a significant prognostic difference. The CTP-A group is traditionally considered to have well-preserved liver function. However, patients in this group had heterogeneous subclinical levels of liver damage, necessitating a precise assessment of the tool. It has been reported that ALBI can provide a more accurate assessment even in the CTP A cohort. The retrospective study by Lo et al. found that 78.3% of the total patients were classified as CTP class A, and using the ALBI grade, this group was stratified into two distinct mortality groups [10]. ALBI grade 1 patients had a median OS of 37.1 months, significantly longer than the 18.6 months observed for ALBI grade 2 patients. However, no significant difference in OS was observed when the CTP A group was further subdivided into CTP A5 and A6. Similarly, ALBI superiority in CTP class A has been reported with other local treatment modalities such as surgical resection and RFA [18]. They reported that CTP class A accounted for 71.8% of all patients and that ALBI grades are more reliable in assessing liver function and predicting OS.

In our study, the optimal ALBI cutoff value to predict post-SBRT CTP increases was −2.47. The cutoff value showed a sensitivity of 85.7% and a specificity of 71.1%. The value was higher than −2.60, the widely accepted reference point for ALBI grades 1 and 2. The ALBI rating was initially applied to a Japanese training set, where patients were classified into different grades. Grade 1 was assigned to the lowest-risk group, representing 25% of patients with the lowest risk of death. Grade 3 was designated for the highest-risk group, consisting of 10% of patients with the highest risk of death. The remaining patients, falling between the lowest and highest risk groups, were categorized as grade 2. Subsequently, the model was validated in patients from various geographical regions. The validation group included patients with HCC treated with resection, sorafenib, and non-HCC cirrhosis [7]. There is limited reporting on the cutoff value for ALBI in HCC SBRT settings. Recently, Lo et al. identified an optimal cutoff [10]. The study analyzed 152 patients with HCC who underwent SBRT, with 78.3% having CTP class A. The study found that the optimal ALBI cutoff value for predicting RILD was −2.76, close to the cutoff point between ALBI grades 1 and 2. The sensitivity and specificity of this cutoff value for predicting clinical RILD were 95% and 32%, respectively. The authors noted that because the sensitivity was high, dose escalation or reirradiation may be considered in patients within the safe zone. However, because of the relatively low specificity of the ALBI score at −2.76, they suggested that CTP class B liver function may be a more appropriate exclusion criterion for clinical trials.

One of the most useful tools for predicting liver complications after resection is the indocyanine green 15-minute retention (ICG-R15) test [19,20,21,22]. The retention rate of ICG after intravenous infusion provides a dynamic, real-time quantitative assessment of liver function. A few studies have correlated pre-RT ICG-R15 with liver complications in patients treated with RT [23,24,25]. Based on a dose-volume histogram study, Kawashima et al. argued that liver volume receiving 30% of the isocenter dose (V30%) and ICG-R15 are important indicators for estimating tolerance to proton RT. In this study, all participants with ICG R15 less than 20% were free from proton-inducing hepatic insufficiency [24]. In a retrospective study by Yoon et al., the correlation between pre-RT ICG-R15 and RILD was more clearly demonstrated in patients with HCC treated with RT. The cutoff value for pre-RT ICG-R15 was 22%. The incidence of RILD in patients with 22% or higher ICG-R15 levels was 40.7%, compared to 3.4% in those with lower levels [23]. However, the ICG-R15 test is invasive and not easily obtained in all patients. Investigators have attempted to correlate ICG-R15 values and ALBI scores and found a strong positive correlation [26]. The distribution of ICG-R15 levels has been investigated in more than 3000 patients with HCC in Japan. The liver function corresponding to ICG-R15 levels <10%, <20%, and <30% was consistent with ALBI scores of −2.623, −2.47, and−2.22, respectively [27]. Based on this, it was postulated that the cutoff of −2.47, suggested by the current analysis, corresponded to the degree of liver damage equivalent to ICG-R15 levels <20%. Consistent with the above-mentioned studies suggesting ICG-R15 <20% as a criterion, we propose using an ALBI score of −2.47 as a more easily accessible marker.

Interestingly, we observed that patients with a PTV greater than 65 mL had a significantly higher incidence of RILD than those with a smaller PTV. Furthermore, we categorized patients into four groups based on their ALBI score and PTV and observed varying rates of hepatotoxicity in each group. These findings underscore the crucial role of factoring in the ALBI score and PTV during treatment planning to mitigate the risk of hepatotoxicity in patients receiving liver tumor radiation therapy.

HCC typically occurs in livers that have suffered chronic damage, and patients often undergo multiple liver-directed treatments such as resection, TACE, and RFA before referring to SBRT. Among several known dosimetric parameters (e.g., mean dose, normal liver volume, and volume spared from a certain dose), MLD appears consistently important for risk estimation [28,29]. The Quantitative Analysis of Normal Tissue Effects in the Clinic (QUANTEC) report and prospective data suggested an MLD limit of 18–20 Gy for 6-fraction SBRT [28,29]. In our cohort, the average MLD was 8.0 ± 2.9 Gy, ranging between 2.2–15.4 Gy; thus, MLD was not a significant factor in toxicity analysis. However, we found that even among patients in the dosimetrically ‘safe zone’, the higher the MLD, the greater the predictive power of the ALBI score compared to the CTP score. With recent progress in developing antiviral therapies, the hepatic function reserve in patients with HCC has steadily improved. Most patients with HCC present with CTP-A. The development of systemic therapies, including molecularly targeted therapy and immunotherapy, has significantly improved overall and progression-free survival [30,31,32]. There is a need for a precise assessment of liver function in this group. Further exploration of the interaction between MLD and ALBI scores is needed for personalized treatment of patients with HCC.

There are some limitations to our study. Firstly, the retrospective design of this study is susceptible to selection bias, although we included all consecutive cases. Secondly, due to the exclusion of CTP class C patients from receiving SBRT, only one patient with ALBI grade 3 was included and considered ALBI grade 2 for statistical analysis. Finally, although our study found the effectiveness of ALBI grade even in patient populations where CTP A was predominant, further investigation is needed to determine its superiority over CTP A5 and A6 and its continued effectiveness within the CTP A5 patient subgroup.

## 5. Conclusions

In patients with HCC after SBRT, the ALBI score at baseline was an independent predictor of hepatotoxicity. The optimal cutoff value of the ALBI score was −2.47, with a sensitivity of 85.7% and a specificity of 71.1%. Within the safe MLD delivery range, the higher the MLD, the greater the predictive power of the ALBI score compared to the CTP score. Further evaluation of the relationship between MLD and ALBI scores is warranted to enhance the precision of personalized treatment strategies for patients with HCC.

## Figures and Tables

**Table 1:** Patient and baseline characteristics.

Variables		n	(%)
Sex	Male	100	(82.6)
	Female	21	(17.4)
Age	mean, year (range)	67.9	(60–76)
Underlying liver disease	HBV	60	(49.6)
	HCV	31	(25.6)
	Others	30	(24.8)
Initial platelet count	Median, k	129.1 ± 54.7	
Child–Pugh Class	A	112	(92.6)
	B	9	(7.4)
Child–Pugh Score	5	93	(76.9)
	6	19	(15.7)
	7	4	(3.3)
	8	5	(4.1)
ALBI score		−2.6 ± 0.4	
ALBI grade	1	66	(54.5)
	2	55	(44.7)
	3	1	(0.8)
Tumor size	median, cm^3^	2.7 ± 1.1	
GTV	median, cm^3^	8.7 ± 8.0	
PTV	median, cm^3^	42.8 ± 22.7	
Radiation dose	36 Gy	2	(1.7)
	40 Gy	16	(13.2)
	44 Gy	4	(3.3)
	48 Gy	99	(81.8)
Normal liver volume	median, cm^3^	1244.9 ± 262.2	
Mean liver dose	median, Gy	8.0 ± 2.9	
V15	median, cm^3^	1023.5 ± 249.6	

Abbreviations: HBV, hepatitis B virus; HCV, hepatitis C virus; ALBI, albumin–bilirubin; GTV, gross target volume; PTV, planning target volume; V15, liver volume receiving a dose of 15 Gy or higher.

**Table 2:** Univariate and multivariable logistic regression for liver toxicity.

	Univariate Analysis			Multivariate Analysis		
Variable	OR	(95% CI)	*p*	OR	(95% CI)	*p*
Sex						
Male	0.503	(0.0752–5.656)	0.350			
Age	0.965	(0.900–1.088)	0.840			
Platelet	0.996	(0.982–1.011)	0.650			
Child–Pugh Score	2.790	(1.399–5.564)	0.004			
Child–Pugh Class						
A						
B	12.828	(1.540–97.824)	0.009			
ALBI Score	13.186	(2.270–76.579)	0.004	29.51	(3.860–418.400)	0.003
ALBI Grade						
1						
2	7.842	(0.907–370.904)	0.046			
Tumor size	1.422	(0.737–2.742)	0.294			
GTV	1.058	(0.987–1.135)	0.115			
PTV	1.040	(1.011–1.070)	0.007	1.05	(1.020–1.090)	0.004
Radiation Dose	0.851	(0.706–1.025)	0.090			
Normal liver volume	1.000	(0.997–1.003)	0.856			
Mean Liver dose	1.151	(0.892–1.485)	0.281			
V15	1.000	(0.996–1.003)	0.756			

Abbreviations: ALBI, albumin–bilirubin; GTV, gross target volume; PTV, planning target volume; V15, liver volume receiving a dose of 15 Gy or higher.

**Table 3:** Univariate and multivariable logistic regression for overall survival.

	Univariate Analysis			Multivariate Analysis		
Variable	HR	(95% CI)	*p*	HR	(95% CI)	*p*
Sex						
Male	0.33	(0.1–1.1)	0.072			
Age	0.99	(0.92–1.06)	0.723			
Platelet	1	(0.99–1.01)	0.667			
Child–Pugh Score	1.95	(1.08–3.53)	0.026	2.793	(1.37–5.71)	0.005
Child–Pugh Class						
A						
B	4.39	(0.91–21.22)	0.066			
ALBI Score	2.69	(0.7–10.34)	0.149			
ALBI Grade						
1						
2	1.61	(0.51–5.11)	0.417			
Tumor size	1.82	(1.11–2.98)	0.017	2.184	(1.23–3.87)	0.007
GTV	1.03	(0.98–1.08)	0.319			
PTV	1.01	(0.99–1.03)	0.286			
Underlying liver disease						
hepatitis B						
hepatitis C	0.96	(0.25–3.74)	0.953			
Others	0.69	(0.14–3.36)	0.647			
Radiation Dose	0.96	(0.8–1.14)	0.617			
Normal liver volume	1.00	(1.00–1.00)	0.258			
Mean Liver dose	1.03	(0.84–1.26)	0.776			
rV15	1.00	(1.00–1.00)	0.347			
AFP	1.00	(1.00–1.00)	0.771			
PIVKA-II	1.00	(1.00–1.00)	0.011	1.00	(1.00–1.00)	0.18

## Data Availability

The data of this work can be disclosed by mail contact with the correspondent and the legal office.
